# Resin Wing Auxiliary Technique for Passive Reattachment of Anterior Crown Fracture Fragments: A Clinical Case Report With 24‐Month Follow‐Up and Clinical Technical Tips

**DOI:** 10.1002/ccr3.73476

**Published:** 2026-09-03

**Authors:** Jian Zhang

**Affiliations:** ^1^ Department of Endodontics The Affiliated Stomatological Hospital of Jiujiang University Jiujiang Jiangxi China

**Keywords:** adhesive restoration, crown fractures, dental trauma, fragment reattachment, resin wing auxiliary technique, vital pulp therapy

## Abstract

Crown fractures often hit maxillary central incisors. Fragment reattachment is ideal yet prone to misalignment via manual bonding. This report introduces a resin wing scaffold for compression‐free precise fragment seating, detailing its clinical steps, 24‐month follow‐up of a fractured crown, alongside mechanical insights and intraoperative risk control.

## 1 Introduction

Traumatic crown fractures represent a frequent clinical presentation in pediatric and general dental practices, with anterior permanent incisors particularly susceptible to injury due to their prominent facial position and incomplete eruption status in adolescent patients [1]. Autologous fragment reattachment is preferentially selected over indirect restorations owing to its superior aesthetic integration, preservation of natural tooth morphology, and ability to sustain pulp perfusion following appropriate vital pulp management [2, 3]. Traditional freehand bonding relies on continuous manual compression to stabilize fragments during composite curing. This clinical workflow frequently leads to three key adverse outcomes: interfacial malalignment, inconsistent luting composite thickness, and residual interfacial stress. These defects act as primary contributors to marginal leakage, premature debonding, and secondary fracture failure of reattached fragments [4, 5, 6].

The Resin Wing Auxiliary Technique (RWT) addresses these clinical limitations by constructing a rigid temporary composite scaffold anchored to adjacent teeth. The scaffold delivers a stable three‐dimensional spatial reference for passive fragment placement without external compressive force applied by the clinician. This case report details the chairside stepwise operation of RWT, records intraoperative risk control interventions, and presents 24‐month clinical and radiographic follow‐up outcomes of a complicated crown fracture managed with RWT‐assisted fragment reattachment. Clinical mechanical observations supporting this auxiliary method and long‐term monitoring results are further discussed.

## 2 Case Report

### 2.1 Case History

An 11‐year‐old male patient presented to the emergency dental clinic with an acute traumatic injury to the maxillary anterior dentition. The detached fractured crown fragment was recovered and stored in whole milk for 7 h prior to clinical intervention. The patient's medical history was unremarkable, with no prior dental trauma or developmental dental anomalies documented.

### 2.2 Preoperative Clinical and Radiographic Assessment

**FIGURE 1:**
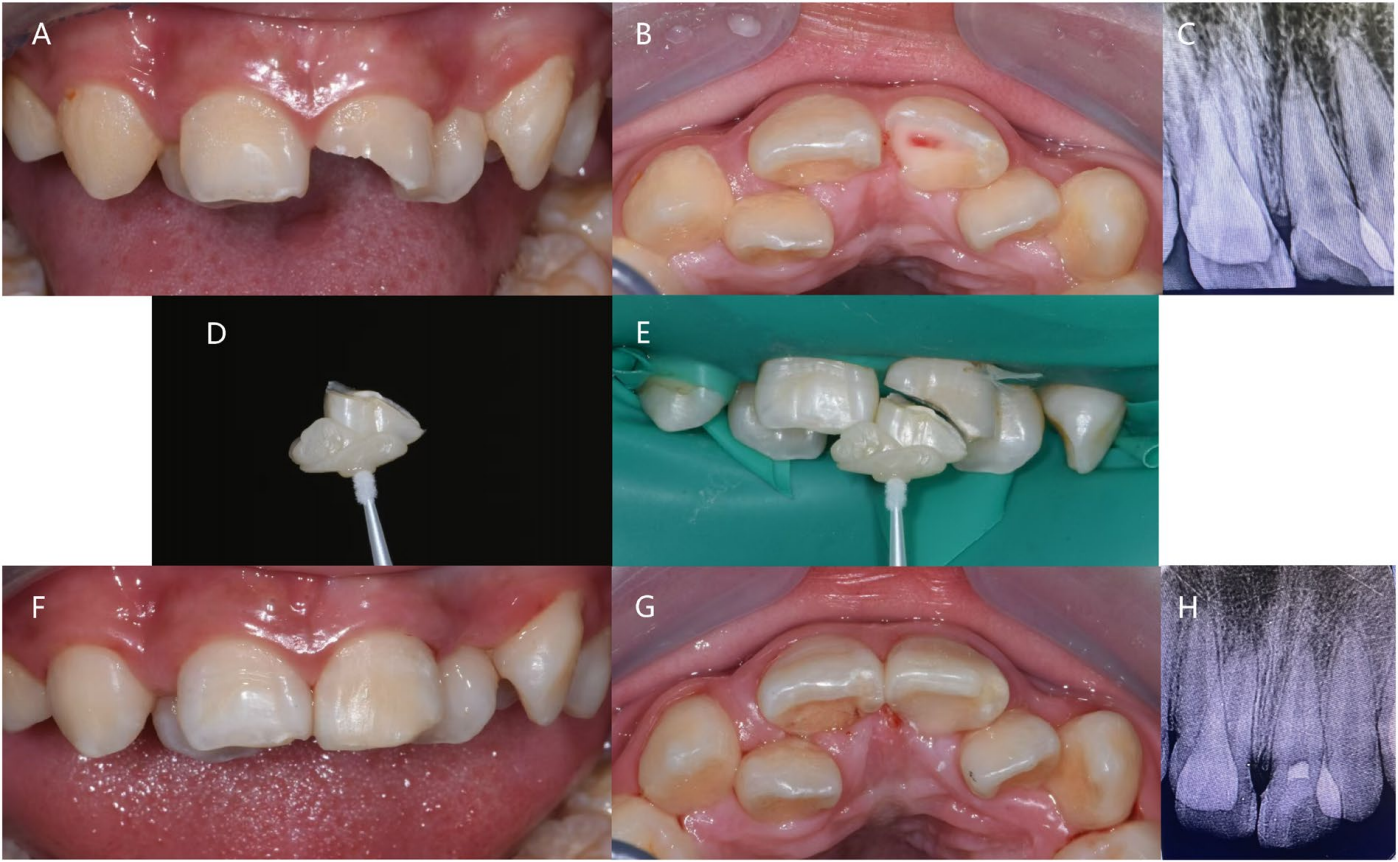
Clinical presentation of an 11‐year‐old male patient with traumatized maxillary anterior teeth; the fractured crown fragment was preserved in milk for 7 h post‐injury. (A, B) Intraoral clinical photographs reveal crowded maxillary anterior dentition. Tooth 11 exhibited mesial incisal enamel loss without pathological mobility. Tooth 21 sustained an oblique crown fracture involving one‐third of the clinical crown, accompanied by a palatal fracture margin extending approximately 0.5 mm subgingivally and pulp exposure. The pulp exposure measured 1 mm in diameter, and the fractured fragment demonstrated a distal incisal defect when anatomically repositioned. (C) Preoperative periapical radiographs. Tooth 11 presented Nolla stage 10 root development, continuous root canal walls, and widened periodontal ligament space. Tooth 21 showed a pulp‐chamber‐involving crown fracture, intact root canal walls, and Nolla stage 10 root maturation with widening of the periodontal ligament space. (D) Following minimally invasive partial gingivectomy with an electrosurgical unit, a custom resin wing was fabricated on the isolated fractured crown fragment. (E) Rubber dam isolation was implemented, and passive seating of the fractured crown with resin wing support was verified intraorally. (F, G) After completion of partial pulpotomy on tooth 21, the fractured crown fragment was passively positioned and bonded with resin wing assistance. A shallow concave bevel was prepared along the labial fracture margin, followed by resin reinforcement, occlusal adjustment, and finishing/polishing of the restoration. (H) Postoperative periapical radiograph displays radiopaque pulp‐capping material at the root canal orifice of tooth 21; the reattached crown fragment remained undisplaced with a uniformly thick adhesive interface.

Intraoral examination revealed crowding of the maxillary incisors (Figure 1A,B). Tooth 11 exhibited localized mesial incisal enamel loss without pathological mobility or pulp exposure. Tooth 21 suffered an oblique complicated crown fracture extending through one‐third of the clinical crown; its palatal fracture margin extended 0.5‐mm subgingivally, accompanied by a 1‐mm pulp exposure. Anatomical reapproximation of the recovered fragment revealed an irregular distal incisal contour defect.

Preoperative periapical radiography (Figure 1C) demonstrated Nolla stage 10 root development in both incisors, intact root canal walls, and diffuse widening of the periodontal ligament space consistent with traumatic dental concussion. The fracture line of tooth 21 fully penetrated the pulp chamber, confirming a complicated crown fracture diagnosis.

### 2.3 Clinical Treatment Sequence

Minimally invasive partial gingivectomy was performed using a high‐frequency electrosurgical unit to expose the subgingival fracture margin of tooth 21 (Figure 1D). The recovered crown fragment was isolated, anatomically aligned, and a custom provisional resin wing was fabricated on its labial surface.

Rubber dam isolation was applied to the maxillary anterior sextant, and passive seating of the fragment supported by the resin wing scaffold was verified intraorally without mechanical compression (Figure 1E).

Partial pulpotomy was performed on tooth 21 per IADT guidelines; Pulpotomy was conducted under the microscope, with direct pulp capping using the bioceramic material iRoot BP Plus (Innovative BioCeramix Inc., Canada), and a base of light‐cured calcium silicate TheraCal LC (Bisco Inc., USA). The bonding interface of the base tooth and fractured tooth segment was etched with 35% phosphoric acid (Heraeus Inc., Germany) and coated with 3 M eighth‐generation universal adhesive (3 M Inc., USA) [3, 7, 8]. The fractured tooth segment was repositioned and bonded to the base tooth using resin of Beautifil flow plus F00 (Shofu Inc., Japan), and the fragment was passively seated via the resin wing fulcrum. Excess interproximal composite was removed, followed by 20‐s full light curing on both buccal and lingual aspects.

A shallow concave bevel was prepared along the labial fracture margin to reinforce interfacial fracture resistance with supplementary flowable composite [9]. Occlusal adjustment eliminated all centric and protrusive premature contacts, and the restoration was sequentially finished and high‐gloss polished (Figure 1F,G).

Postoperative periapical radiography confirmed uniform adhesive layer thickness, stable fragment positioning, and radiopaque bioceramic pulp‐capping material at the root canal orifice of tooth 21 (Figure 1H).

### 2.4 Twenty‐Four‐Month Follow‐Up Evaluation

**FIGURE 2:**
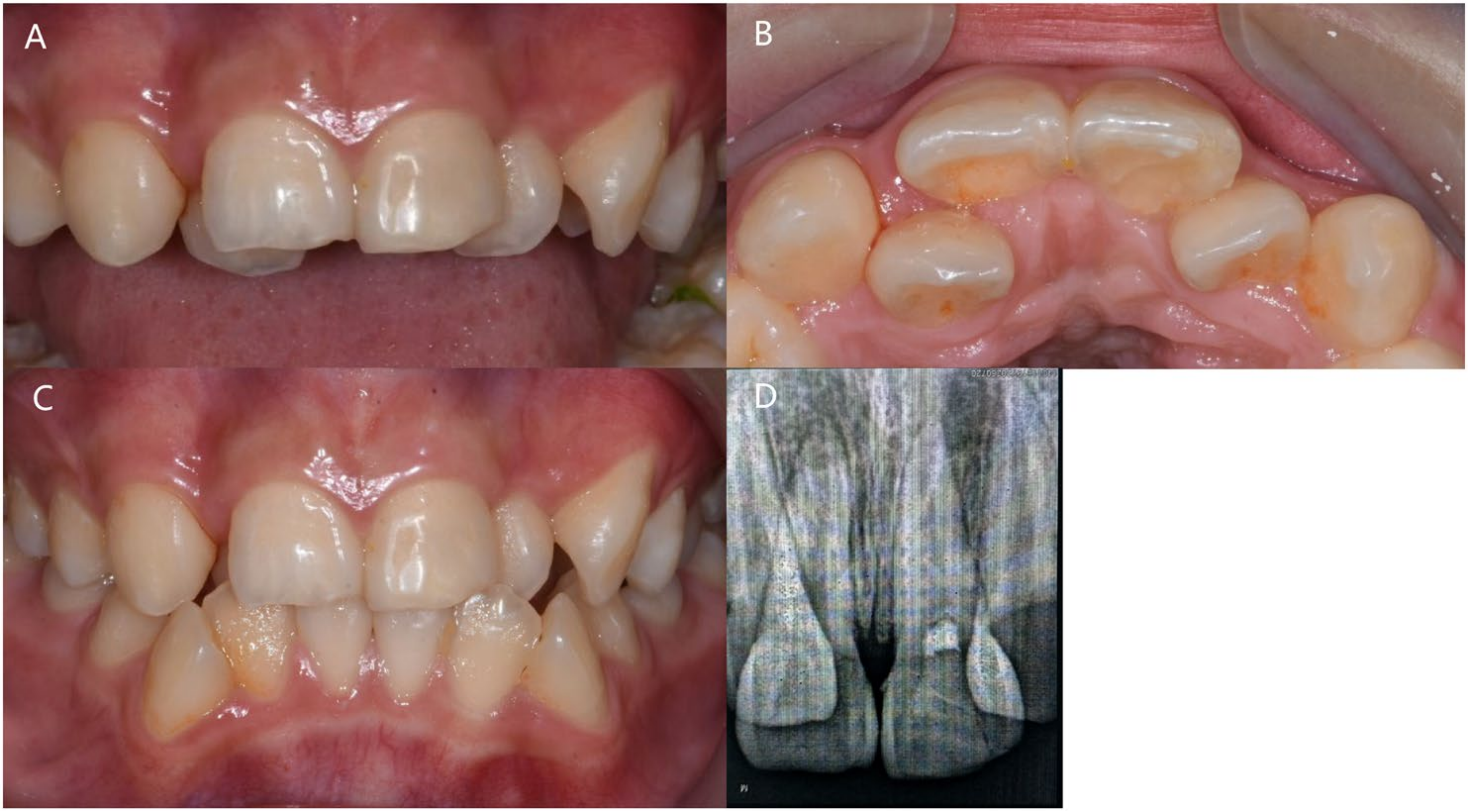
(A–C) Twenty‐four‐month postoperative follow‐up images of tooth 21 show stable tooth anatomy. Positive electric pulp test responses were recorded, with stimulation thresholds consistent with the contralateral central incisor; no hypersensitivity or delayed pain was observed, verifying intact pulp sensory function. (D) A 24‐month follow‐up periapical radiograph demonstrates a fully formed calcific bridge at the pulp capping site over the root canal orifice of tooth 21.

At the 24‐month recall appointment, intraoral photographs demonstrated stable anatomical contour of tooth 21 with no marginal discoloration, wear, or fragment separation (Figure 2A–C). Electric pulp testing elicited threshold responses comparable to the contralateral central incisor; no spontaneous pain, cold hypersensitivity, or sinus tract formation was recorded, confirming sustained pulp viability [8, 10].

Follow‐up periapical radiography revealed a continuous, intact calcific bridge at the pulp‐capping site over the root canal orifice of tooth 21, with normalized periodontal ligament width and no evidence of periapical radiolucency (Figure 2D) [8, 11].

## 3 Two‐Step Clinical Operation of the Resin Wing Auxiliary Technique

RWT operates via two sequential bonding phases to eliminate active seating artifacts and standardize adhesive layer geometry: the scaffold fabrication phase and passive fragment luting phase. Upon complete composite polymerization, the temporary resin wing is detached to restore native tooth anatomy. The chairside clinical workflow is described below:

### 3.1 Step 1: Resin Wing Fabrication and Anatomical Pre‐Positioning

After precise anatomical reapproximation of the fractured crown fragment, flowable composite is applied to the labial fracture margin for temporary fixation. A minimal volume of adhesive is applied to the incisal edge, followed by coverage of the incisal edge and partial labial surface with composite resin. The composite is extended toward the adjacent tooth to construct the resin wing, with a minimum wing thickness of 1.5 mm maintained throughout the structure. A rigid stabilizing pivot is formed following a 10‐s light‐curing cycle.

### 3.2 Step 2: Passive Fragment Seating and Adhesive Luting

Universal adhesive systems are uniformly utilized for surface pretreatment of both fractured crown fragments and residual abutment tooth substrate. Flowable luting composite is applied to the fracture interface, and the resin wing scaffold enables non‐compressive passive adaptation of the fragment. Excess interproximal composite is cleared, and 20‐s light irradiation is delivered to both buccal and lingual surfaces to achieve complete polymerization.

### 3.3 Step 3: Resin Wing Removal and Restorative Finishing

Resin wings were detached using a dental excavator. Remnant composite material was thoroughly curetted with a 12D scalpel blade, and fracture margins were refined with fine dental burs prior to supplementary resin reinforcement of the tooth structure [9]. Comprehensive occlusal adjustment was performed to eliminate all occlusal interferences, followed by high‐gloss polishing of the entire restored tooth surface.

## 4 Discussion

Fragment reattachment remains the most biologically conservative treatment for traumatized anterior crowns, yet technical inconsistencies during fragment positioning limit long‐term restoration survival in complex injury presentations [12]. Conventional bonding techniques rely on operator hand pressure to hold fragments in place during composite curing, which frequently introduces uneven adhesive layer thickness and residual interfacial stress, accelerating marginal leakage and debonding failure over time.

The Resin Wing Auxiliary Technique overcomes these technical limitations by separating fragment anatomical alignment from continuous manual compression, relying on a mechanically stabilized composite scaffold for passive positioning. From a clinical mechanical perspective, passive seating eliminates artificial compressive deformation of fracture interfaces, while the rigid wing stabilizes fragment position to preserve uniform adhesive layer thickness—two critical clinical factors associated with improved long‐term bond durability for traumatic fragment reattachment restorations.

For complicated crown fractures with pulp exposure, integration of IADT‐recommended partial pulpotomy with bioceramic pulp capping prior to fragment reattachment preserves pulp vitality and facilitates continued root maturation in adolescent permanent teeth, as demonstrated by complete calcific bridge formation observed at 24‐month follow‐up in the present case. Minimally invasive electrosurgical gingivectomy avoids the soft tissue scarring and recession risks associated with flap surgery for subgingival fracture margins, maintaining optimal aesthetic gingival architecture in the anterior aesthetic zone.

Several intraoperative complications and corresponding preventive strategies are summarized based on clinical experience with RWT:

1. Difficult resin wing removal caused by unintended adhesion to adjacent teeth or mechanical locking within interproximal undercuts: Isolate the target tooth preoperatively; limit micro‐bonding spots only to the incisal edge of the fractured tooth and avoid sculpting composite into interproximal undercut regions during resin wing fabrication.
2. Incomplete fragment seating and interfacial maladaptation induced by soft tissue obstruction or morphological changes after vital pulp therapy: Fully expose all fracture surfaces, complete rubber dam isolation of the operative field, and conduct trial passive seating of the resin wing scaffold in advance. Selective minor grinding of residual tooth substrate enables full passive adaptation of the fragment.
3. Postoperative fragment debonding triggered by occlusal interferences: Complete comprehensive centric and protrusive occlusal adjustment immediately after restoration to eliminate all premature occlusal contacts.
4. Marginal refracture caused by excessively thin residual dentinal walls and subclinical microcracks: Prepare a shallow chamfer bevel along the fracture margin before supplementary composite reinforcement to strengthen the adhesive‐dentin interface and improve overall fracture resistance.

This auxiliary technique can be extended to multiple trauma categories beyond pulpal‐exposed complicated crown fractures, including uncomplicated crown fractures with intact pulp and mildly mobile luxated incisors requiring temporary splinting support.

Key limitations of RWT include reliance on a structurally intact adjacent tooth to support the provisional resin wing, precluding its use in patients with bilateral multi‐tooth trauma or severely compromised proximal tooth structure. Further prospective longitudinal clinical trials with larger sample cohorts are required to validate the generalizability of RWT across all permanent dentition traumatic injury subtypes and establish evidence‐based survival data relative to conventional fragment reattachment protocols.

Strict adherence to defined clinical indications, rigorous intraoperative isolation and adhesive standardization, and structured long‐term postoperative pulp and periodontal surveillance are indispensable to maximize RWT treatment success.

## 5 Conclusions

The Resin Wing Auxiliary Technique is a clinically practical auxiliary modification for conventional crown fragment reattachment, eliminating fragment displacement induced by operator manual compression and maintaining uniform adhesive layer thickness during restoration curing. This chairside clinical tip delivers predictable aesthetic and functional outcomes for multi‐fragment crown fractures and shallow subgingival anterior traumatic injuries, and can additionally provide temporary splinting support for mildly mobile traumatized incisors. When combined with routine vital pulp preservation therapy and strict operative contamination control, this minimally invasive auxiliary method enables durable, biologically conservative rehabilitation of traumatic crown injuries in adolescent permanent incisors.

## Author Contributions

**Jian Zhang:** conceptualization, methodology, data curation, writing – review and editing, writing – original draft, investigation, validation, formal analysis, project administration, visualization, funding acquisition, resources, supervision.

## Funding

This clinical case report was supported by a research grant from the Affiliated Stomatological Hospital of Jiujiang University.

## Ethics Statement

All clinical procedures were performed in full accordance with international clinical dental research guidelines. Written informed consent was obtained from the patient's legal guardian prior to treatment, data collection, and radiographic documentation.

## Consent

Separate written informed consent for the publication of anonymized clinical intraoral photographs and radiographs was obtained from the patient's legal guardian.

## Conflicts of Interest

The author declares that financial support for this research/case report was provided by The Affiliated Stomatological Hospital of Jiujiang University. The funding source had no involvement in study design, data collection/analysis, interpretation of results, manuscript writing, or the decision to submit for publication. The author affirms that there are no additional financial, personal, or professional relationships that could be construed as influencing the objectivity of this work. The content and conclusions are solely the responsibility of the author and reflect independent academic judgment.

## Data Availability

Raw, anonymized clinical photographic and radiographic data supporting the findings of this study are available from the corresponding author upon reasonable request for non‐commercial research use.
